# The Dynamics of Somatic Mutagenesis During Life in Humans

**DOI:** 10.3389/fragi.2021.802407

**Published:** 2021-12-17

**Authors:** Freek Manders, Ruben van Boxtel, Sjors Middelkamp

**Affiliations:** Princess Máxima Center for Pediatric Oncology and Oncode Institute, Utrecht, Netherlands

**Keywords:** somatic mutation, mutation accumulation, non-malignant cells, aging, fetal development, mutational signatures, genomics

## Abstract

From conception to death, human cells accumulate somatic mutations in their genomes. These mutations can contribute to the development of cancer and non-malignant diseases and have also been associated with aging. Rapid technological developments in sequencing approaches in the last few years and their application to normal tissues have greatly advanced our knowledge about the accumulation of these mutations during healthy aging. Whole genome sequencing studies have revealed that there are significant differences in mutation burden and patterns across tissues, but also that the mutation rates within tissues are surprisingly constant during adult life. In contrast, recent lineage-tracing studies based on whole-genome sequencing have shown that the rate of mutation accumulation is strongly increased early in life before birth. These early mutations, which can be shared by many cells in the body, may have a large impact on development and the origin of somatic diseases. For example, cancer driver mutations can arise early in life, decades before the detection of the malignancy. Here, we review the recent insights in mutation accumulation and mutagenic processes in normal tissues. We compare mutagenesis early and later in life and discuss how mutation rates and patterns evolve during aging. Additionally, we outline the potential impact of these mutations on development, aging and disease.

## Introduction

Virtually every cell in the body contains a unique set of changes to the genome due to the accumulation of somatic mutations during life. Some of the mutations cells acquire during life can contribute to the development of age-associated diseases, such as cancer (Martincorena and Campbell, 2015). Mutations can result from errors made during DNA replication or from unrepaired or incorrectly repaired DNA damage. Each mutational process leaves characteristic patterns of mutations, or “mutational signatures”, in the genome, which can be identified by systematically studying mutation spectra (Alexandrov et al., 2013; Koh et al., 2021).

Somatic mutations have historically been hard to detect, because they are often present in only a tiny fraction of an individual’s cells resulting in a low variant allele frequency (Dou et al., 2018). As a result, most somatic variants are not detected by regular bulk tissue sequencing technologies. Notable exceptions to this are somatic variants in cancer. Since cancers grow out from a single cell, all the somatic variants in that original cell will be clonally present in the cancer. Somatic mutations in cancer have been extensively studied (ICGC/TCGA Pan-Cancer Analysis of Whole Genomes Consortium, 2020). However, to better understand which somatic mutations and mutational processes contribute to cancer, somatic mutations in cancer need to be compared to somatic mutations in normal, pre-cancer tissues.

In the last few years, technological developments in DNA sequencing methods and bioinformatic approaches have enabled the detailed study of somatic mutations in normal tissues. *In vitro* expansion of a single stem cell, followed by whole genome sequencing of the clone, enables highly accurate characterization of the genome of a single cell (Welch et al., 2012). Additionally, somatic variants can be identified by (deep) sequencing of natural occurring clonal patches in healthy tissues using low-input sequencing (Ellis et al., 2021). A disadvantage of these methods is that they are limited to cells with self-renewal capacity. Direct single-cell sequencing after whole genome amplification and single-molecule duplex sequencing are methods that can also be applied to non-dividing cells. Until recently these methods had a relatively low accuracy in mutation detection, but new studies using novel technical and bioinformatic innovations claim to have significantly reduced their error rates (Abascal et al., 2021; Luquette et al., 2021; Xing et al., 2021).

Many studies have recently applied these techniques to characterize somatic mutation accumulation in various tissues of healthy human donors across a wide range of ages. Here, we provide an overview of the findings of these papers and discuss how somatic mutagenesis evolves during life. First, we review how mutations accumulate linearly with age and how this differs between tissues in adults. Building on this knowledge of mutagenesis in adults, we show how mutagenesis is divergent early in life before birth. Finally, we discuss the impact of somatic mutations and how this is different between mutations that occur early and later in life.

### Adult Tissues Accumulate Mutations Linearly With Age

**FIGURE 1:**
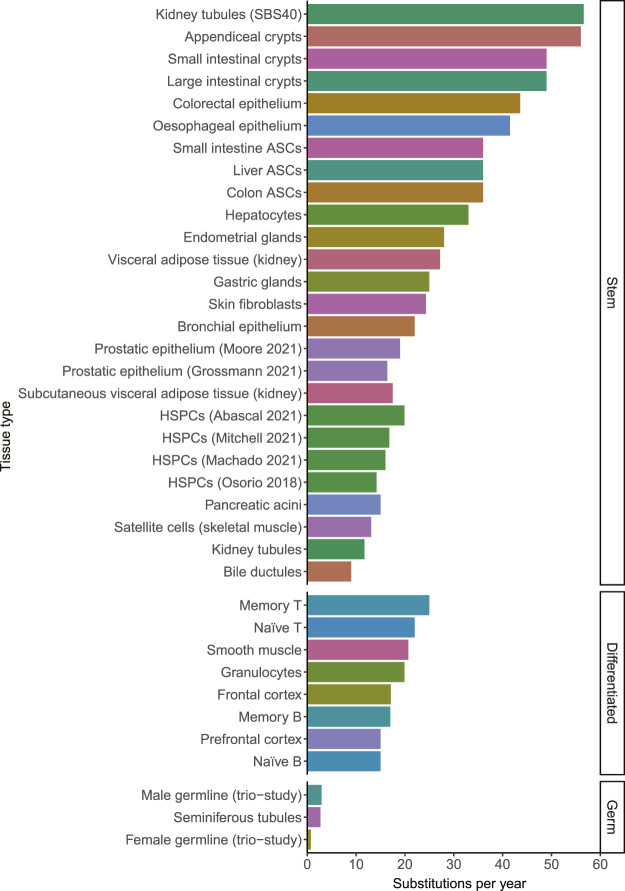
The number of substitutions per year for different tissue types. When the mutation rate of a tissue has been determined multiple times, they are distinguished by the last name of the first author and the publication year. The color indicates the tissue type. The mutation rates in this figure may be influenced by technical differences between the studies, which may explain some of the small differences between tissues. HSPCs; Hematopoietic stem and progenitor cells. ASCs; adult stem cells. Source of mutation rates: Kidney tubules, Subcutaneous visceral adipose tissue (kidney) and Visceral adipose tissue (kidney) (Franco et al., 2019). Appendiceal crypts, small intestinal crypts, large intestinal crypts, gastric glands, pancreatic acini, bile ductules and seminiferous tubules (Moore et al., 2021). Colorectal epithelium (Lee-Six et al., 2019) Esophageal epithelium (Yokoyama et al., 2019). Small intestine ASCs, liver ASCs and colon ASCs (Blokzijl et al., 2016). Hepatocytes (Brunner et al., 2019). Endometrial glands (Moore et al., 2020). Skin fibroblasts (Park et al., 2021). Bronchial epithelium (Yoshida et al., 2020). Prostatic epithelium (Grossmann et al., 2021; Moore et al., 2021). HSPCs (Osorio et al., 2018; Abascal et al., 2021; Machado et al., 2021; Mitchell et al., 2021). Satellite cells (Franco et al., 2018). Memory T, Naïve T, Memory B, Naïve B (Machado et al., 2021). Smooth muscle, granulocytes, Frontal cortex (Abascal et al., 2021). Prefrontal cortex (Luquette et al., 2021). Male and female germlines (Jónsson et al., 2017).

A mutation that is acquired in 1 cell, will be propagated to all of its progeny. It has become clear in recent years that the somatic mutation burden increases remarkably linearly with age in single cells in normal tissues (Blokzijl et al., 2016). So far, this linear mutation accumulation is confirmed in stem cells of all studied normal tissues including liver, small intestine, large intestine, lung, skin, blood, esophagus, muscle, kidney, adipose tissue, endometrium, bile duct, stomach, prostate, pancreas, appendix and bladder (Blokzijl et al., 2016; Franco et al., 2018; Lee-Six et al., 2018; Osorio et al., 2018; Brunner et al., 2019; Franco et al., 2019; Lee-Six et al., 2019; Yokoyama et al., 2019; Lawson et al., 2020; Moore et al., 2020; Yoshida et al., 2020; Grossmann et al., 2021; Mitchell et al., 2021; Moore et al., 2021; Park et al., 2021). The amount of mutations that accumulate in different tissues ranged from 9 substitutions per year in bile ductular cells to 56 substitutions per year in appendiceal crypts (Moore et al., 2021). Mutation rates in other tissues fell within this range, showing that while there are differences between tissues, they all fall within a single order of magnitude (Figure 1). Female and male germ cells acquire only 0.74 and 2.7 mutations per year, showing that the mutation rate in somatic cells is much higher than in germline cells (Rahbari et al., 2016; Jónsson et al., 2017; Moore et al., 2021). The average mutation rates within tissues appear to be relatively constant during adult life. However, individual cells may have mutation burdens divergent from the average burden in the tissue due to different exposures to endogenous or exogenous mutagenic processes and due to differentiation, as will be discussed in the next sections.

### Tissue-specific Mutational Processes in Adult Stem Cells

Mutational signature analysis has indicated that some mutagenic processes are active in all tissues, whereas some are tissue- or exposure-specific. Single base substitution signature (SBS) 1 and SBS5, which reflect life-long activity of “clock-like” mutational processes, which cause mutations at a steady rate, were found in all cell types. SBS1 mutations are thought to be caused by spontaneous deamination of methylated cytosine residues into thymine. In contrast, the cause for SBS5 mutations is unknown, but likely represents a collective of endogenous background mutational processes (Nik-Zainal et al., 2012; Alexandrov et al., 2013). While most, if not all, tissues gradually accumulate SBS1 and SBS5 mutations throughout life, their ratios differ between tissues. Differences in cell turnover rate between tissues have been suggested to be one possible cause for this (Alexandrov et al., 2015; Blokzijl et al., 2016; Li et al., 2021; Moore et al., 2021).

Some cells also showed contributions of additional mutational signatures caused by both exogenous and endogenous factors, which explain part of the variation in the mutation rate and spectra between tissues. Most skin fibroblasts and melanocytes, for example, show contribution of SBS7, a signature caused by UV-radiation (Tang et al., 2020; Park et al., 2021). Similarly, kidney tubule cells with contribution of SBS40, in this case possibly caused by formaldehyde and alkylating agents, were found to accumulate 56.6 SNVs per year whereas cells lacking this signature only accumulated 11.7 SNVs per year (Franco et al., 2019). SBS16, which is associated with alcohol consumption, could be found in cells of the esophagus (Yokoyama et al., 2019; Moore et al., 2021). Colibactin produced by a specific common *E. coli* strain was found to cause SBS88 mutations in some colon crypts (Lee-Six et al., 2019; Pleguezuelos-Manzano et al., 2020; Moore et al., 2021). SBS2 and SBS13, which are associated with activity of endogenous APOBEC cytosine deaminases, have been found in multiple cell types including lung, colorectal and small intestinal cells (Lee-Six et al., 2019; Yoshida et al., 2020; Moore et al., 2021). These signatures are caused by sporadic bursts of mutagenesis in a subset of cells in a tissue (Petljak et al., 2019). Overall, it is clear that SBS1 and SBS5 are present in almost all cell types and that additional tissue-specific mutational processes can result in an increased mutational burden.

### Mutagenesis in Post-Mitotic and Differentiated Cells

It is likely that there are differences in mutagenesis between stem cells and non-dividing, differentiated cells. Stem cells could be expected to be protected from mutagenesis, because they are long-lived and can self-renew in order to regenerate tissues throughout life, which is not the case for most post-mitotic or fully differentiated cells (Wyles et al., 2014). On the other hand, post-mitotic cells will not accumulate errors made during DNA replication. So far, somatic mutations have been mostly characterized in proliferating stem and progenitor cells due to technical limitations. In the last few years, technical innovations have also enabled a more accurate detection of somatic variants in non-dividing cells. One study using single-cell whole genome amplification found a higher mutation rate in differentiated hepatocytes compared to liver stem cells (Brazhnik et al., 2020). Differentiated granulocytes were found to have a slightly, not significantly, increased mutational load compared to hematopoietic stem cells (Abascal et al., 2021). In contrast, both naïve and memory T-lymphocytes showed an increased mutation rate of 22 and 25 SNVs per year compared to the 16 SNVs per year that this study found in hematopoietic stem cells. Additionally, both memory B- and T-lymphocytes showed an increased mutation load irrespective of age, likely caused by somatic hypermutation, while naïve lymphocytes did not (Zhang et al., 2019; Machado et al., 2021). Interestingly, several studies found that post-mitotic brain neurons accumulate mutations with age at a similar rate (14.7–17.1 per year) as stem cells of other tissues, even though they do not replicate (Lodato et al., 2018; Abascal et al., 2021; Luquette et al., 2021).

Most somatic mutations in short living differentiated cells are likely acquired in the stem cell ancestors of these cells. Their relatively short lifespan might prevent differentiated cells from building up a strongly elevated mutation burden after differentiation, even if their mutation rate would be much higher. In addition to an increased mutation rate, it is also possible that the process of differentiation itself could lead to a single burst of mutation accumulation. Estimating the precise mutation rate *in vivo* differentiated cells is therefore challenging. Overall, the first single-cell and single-molecule sequencing studies on differentiated and post-mitotic cells, suggest that the mutation burden in these cells is either not or only modestly increased as compared to stem cells in the same tissues.

### Somatic Mutation Rates Are Strongly Elevated in Prenatal Cells

Stem cells of adults and even of infants acquire somatic mutations at an apparent constant rate. Interestingly, it has recently become clear that the somatic mutation rate during fetal development is strongly increased compared to the post-natal rates. For example, in hematopoietic stem cells of newborns an average of 40 somatic SNVs were found 9 months after conception, while only 14–17 SNVs are gained in adult stem cells per year (Osorio et al., 2018; Mitchell et al., 2021). This increased mutation rate before birth was confirmed by genomic analyses of various fetal tissues (Bae et al., 2018; Kuijk et al., 2019; Hasaart et al., 2020; Spencer Chapman et al., 2021). The mutation rate in fetal liver, intestine and hematopoietic stem cells was shown to be fivefold higher as compared to the rate in adult tissues. The mutation rate in fetal mitotic neuronal progenitor cells was even suggested to be 50-fold higher than in other postnatal tissues (Bae et al., 2018), although more recent studies could not confirm this high mutation rate (Abascal et al., 2021; Luquette et al., 2021).

**FIGURE 2:**
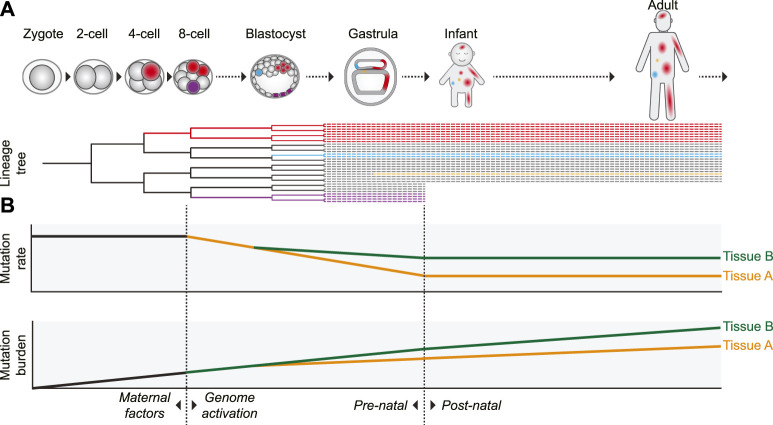
Proposed model of the dynamics of somatic mutagenesis during life. Schematic overview depicting the distributions and rates of somatic mutations. **(A)** Mutations arising early in development can be propagated to many cells of multiple tissues, as indicated by the red cell lineage. Due to this wide distribution, mutations arising early in life can have a strong potential impact on development and disease. Mutations acquired later in life are usually only inherited by a small number of cells (the blue- and orange-colored cells). Some early mutations, depicted by the purple lineage here, may also end up in extra-embryonic cell lineages not contributing to the embryo proper. **(B)** The somatic mutation rate is especially high in the first embryonic cell divisions. After genome activation, the mutation rate decreases. It is unclear if this decrease is gradual (as depicted) or more abrupt, but the mutation rate probably remains relatively high compared to the postnatal mutation rate. After birth, the somatic mutation rate appears to stay remarkably constant during aging, leading to a gradual linear mutation accumulation. Variance in the mutation rate between tissues leads to a tissue-specific mutation burden. In some tissues (such as intestine), the tissue-specific mutation patterns already arise early in embryogenesis, whereas in others (such as liver) these patterns start to emerge only after birth.

Additional evidence for an elevated prenatal mutation rate was provided by retrospective lineage tracing studies of cell lineages in various tissues. Since a mutation that arose in 1 cell will be inherited by all its progeny, somatic mutations can be used as genetic barcodes, which enables the reconstruction of phylogenies and the identification of early embryonic cell divisions (Figure 2) (Baron and van Oudenaarden, 2019). By assessing mutations that are shared between different cells of the same individual it was shown that especially in the first two to 3 cell divisions (up to the 8-cell stage) the mutation rate is relatively high with roughly 2 to 3 mutations per cell division (Ju et al., 2017; Park et al., 2021; Spencer Chapman et al., 2021). In addition to the high mutation rate in the first embryonic divisions, it was previously reported that chromosomal instability, characterized by high levels of chromosomal missegregation and abnormalities leading to mosaicism, is common in early (cultured) human embryos (Vanneste et al., 2009).

Several non-exclusive factors contributing to the high mutation rates in early human embryos have been proposed. The first cell cycles after fertilization are relatively fast, leaving little time for proper DNA repair (Palmer and Kaldis, 2016). Transcription is not yet active in the first cell divisions. This precludes high-fidelity transcription-coupled DNA repair and makes DNA repair entirely dependent on maternally inherited factors, which are diluted with every cell division (Schulz and Harrison, 2019). DNA damage can be caused by chromatin remodeling after fertilization or may be inherited from the sperm cell (Colaco and Sakkas, 2018). Cell cycle and DNA damage checkpoints are relaxed and apoptosis is prevented, raising tolerance to DNA damage and mutagenesis (Palmer and Kaldis, 2016; McCoy, 2017; Vazquez-Diez and FitzHarris, 2018). After the 8-cell stage, coinciding with activation of transcription, the mutation rate drops to less than 1 mutation on average per cell division (Park et al., 2021; Spencer Chapman et al., 2021). The elevated mutation rate, leading to the presence of dozens of somatic mutations in each cell at birth, may be the cost of the rapid growth required during embryonic development (Figure 2B).

### Divergence of Tissue-Specific Mutation Patterns Early in Life

The mutation patterns and rates that are specific for each tissue in adults must emerge at a certain, currently unclear, moment during development. As both cellular functions and exposure to exogenous agents are different before and after birth, it can be expected that mutation accumulation also differs between those phases in life. The earliest embryonic mutations show clock-like mutational signatures that are also common in most adult tissues, namely SBS1 and SBS5 (Ju et al., 2017; Park et al., 2021; Spencer Chapman et al., 2021). Later during development, it has been shown that fetal intestinal cells show the same specific mutation patterns as in adult intestinal cells already at 13 weeks after fertilization (Kuijk et al., 2019). In contrast, liver stem cells and hematopoietic stem cells show different mutation patterns before and after birth (Kuijk et al., 2019; Hasaart et al., 2020). Fetal liver stem cells show a high contribution of SBS18, which is linked to oxidative stress-induced mutagenesis and interestingly was also found at high levels in fetal neural progenitors (Pilati et al., 2017; Bae et al., 2018; Kucab et al., 2019). These findings show that mutation patterns start to diverge between tissues already early in development, but the precise moment appears to be tissue specific.

### The Impact of Somatic Mutations on Disease and Aging

Somatic mutations can influence disease and aging in multiple ways. The most well-known impact of somatic mutations is their involvement in cancer (Hanahan and Weinberg, 2011). Somatic mutations can also lead to non-cancerous, but potentially harmful clonal outgrowth, with clones sometimes replacing entire tissues, for example in clonal hematopoiesis (Jaiswal and Ebert, 2019; Kakiuchi and Ogawa, 2021; Mustjoki and Young, 2021). As has been recently reviewed in detail, accumulation of somatic mutations might also impact aging for example by affecting the functioning of cells by influencing tightly controlled gene-regulatory networks and increasing cell-to-cell transcriptional heterogeneity (transcriptional noise) (Vijg and Dong, 2020). This is further supported by the observation that the mutation load at the end of life is similar between different mammals with wildly different lifespans (Cagan et al., 2021). Defects in DNA repair have also been associated with accelerated aging (Tiwari and Wilson, 2019). However, it has recently been shown that a massively increased somatic mutation rate, in this case due to germline *POLE/POLD1* mutations, does not necessarily lead to accelerated aging (Robinson et al., 2021). Further studies comparing young and old tissues are required to elucidate the complicated effects of somatic mutations on normal and aberrant aging.

### Early Mutations: Small Numbers, but Large Effects

In adults, only a relatively small fraction of all mutations in each cell was acquired prenatally during early development. While these early mutations are less numerous, they are shared by a high fraction of the individual’s cells (Figure 2A) (Behjati et al., 2014; Ju et al., 2017). As a result, some of these mutations might be clinically relevant, as somatic mosaicism can underlie genetic diseases and cancer (Biesecker and Spinner, 2013; Campbell et al., 2015; Fernández et al., 2016; Priest et al., 2016). For example, somatic mosaicism has been associated with autism spectrum disorders as well as other neurological disorders (Poduri et al., 2013; McConnell et al., 2017; Paquola et al., 2017). As many of these disorders originate early in life, especially mutations arising in the earliest phases of development may have a pathogenic impact (Hodges et al., 2020).

A cancer driver mutation arising early in development can be propagated to a large fraction of cells. As a result, a relatively large population of cells will be vulnerable to developing into a malignancy *via* further hits. Consistent with this, driver mutations have been found that originated decades before the development of cancer, some of which likely emerged during fetal development or early childhood (Gerstung et al., 2020; Williams et al., 2020). In addition to adult cancers, pediatric cancers are likely often caused by somatic mutations occurring during development (Filbin and Monje, 2019). It has been shown that 1% of newborns already have detectable driver genomic rearrangements in some of their blood cells, but these only lead to cancer in a very small minority of cases (Greaves, 2018).

Early mutations may also impact spontaneous abortions. Less than half of all human conceptions are thought to lead to a live birth. This is at least partly due to somatic aneuploidies and copy number changes occurring in the first cell divisions, but it is not unlikely that in some cases SNVs also play a role (Hardy and Hardy, 2015).

Mutations that occur early in development can end up in the germline and thereby propagate as *de novo* germline variants in a person’s offspring. Early mutations occurring during development can likely explain a sizable fraction of *de novo* germline variants, because of the low mutation rate of germline cells (Yang et al., 2021). These *de novo* germline variants can cause (neuro-)developmental disorders and other diseases (Veltman and Brunner, 2012; Deciphering Developmental Disorders Study, 2017; Iakoucheva et al., 2019).

## Discussion

Recent studies have revealed that within a healthy tissue the accumulation of somatic mutations in stem cells occurs at a remarkably constant rate during life. Despite significant differences in function, cell turnover, exposures to mutagens and cancer incidence, but also technical differences between studies, the variation in mutation rates and patterns between stem cells of different tissues is surprisingly modest. Most somatic mutations in stem cells of normal tissues are characterized by different contributions of mutational signatures SBS1 and SBS5, but some cells show contributions of signatures caused by tissue-specific endogenous and exogenous exposures. The mutation burden and in many cases also the clonality of tissues increases with aging, but there seem to be no apparent differences in mutation rates and patterns between stem cells of old and young individuals. The elevated mutation rate before birth forms a notable exception (Figure 2). It appears that the rapid growth during fetal development comes at the cost of decreased genomic stability.

Differentiation, which can change the cell’s functions, self-renewal capacity and potentially also the exposure to mutagens, could be expected to lead to changes in mutation rates and spectra. So far, the first genomic studies of differentiated cells suggest that the effect of differentiation on mutagenesis is only modest, possibly due to the relatively short lifespan of differentiated cells. More single-cell studies of both *in vivo* and *in vitro* differentiated cells of various tissues are required to elucidate the precise role of differentiation on mutation accumulation. Intriguingly, post-mitotic neurons also accumulate mutations at a linear rate similar to stem cells of other tissues. This shows that cell division and DNA replication do not necessarily have to be the main drivers of somatic mutagenesis in all cells. The linear mutation accumulation suggests that mutagenesis in these non-dividing cells, likely caused by endogenous DNA damage followed by erroneous DNA repair, also occurs at a relatively steady rate.

Somatic mutations can result in cancer and play a role in other diseases. They have also been associated with aging, though more research is needed to strengthen these claims. It seems likely that mutations arising early in life, even though they are less numerous than somatic mutations occurring during adulthood, regularly impact disease. The rapidly increasing amount of genomics data will help to further elucidate the relative impact of these early-life mutations compared to the ones arising later in life.
